# Pitfall of false localization in basal temporal epilepsy: A clinical vignette

**DOI:** 10.1002/epd2.70121

**Published:** 2025-10-21

**Authors:** Mathieu Dhoisne, Romain Carron, Fabrice Bartolomei, Stanislas Lagarde

**Affiliations:** ^1^ Department of Clinical Neurophysiology Lille University Hospital Lille France; ^2^ INSERM U1172, LilNCog – Lille Neuroscience & Cognition Lille France; ^3^ APHM, Epileptology Department Timone University Hospital Marseille France; ^4^ APHM, Medico‐Surgical Unit of Epileptology and Functional and Stereotactic Neurosurgery and Gamma Knife Radiosurgery Timone University Hospital Marseille France; ^5^ INS, Institut de Neurosciences Des Systèmes, Aix Marseille Université, INSERM Marseille France

False lateralization of ictal EEG onset is uncommon in temporal lobe epilepsy, but has been reported in mesial TLE with hippocampal sclerosis or vascular malformation.^1^, ^2^, ^3^, ^4^, ^5^, ^6^, ^7^ This phenomenon may relate to low‐amplitude ictal discharges from cortical atrophy or cerebral damage, severe hippocampal sclerosis (“burned‐out” hippocampus), rapid contralateral spread via hippocampal commissures, anterior commissure, or frontal limbic pathways, or the coexistence of multiple epileptogenic foci.^1^, ^2^, ^8^, ^9^, ^10^ This clinical vignette highlights the particular value of stereoelectroencephalography (SEEG) for anatomo‐electro‐clinical correlations when ictal EEG onset conflicts with imaging or semiology.

We report a 21‐year‐old right‐handed woman, with no prior medical history, who developed seizures at age nine. They initially consisted of isolated loss of awareness. At age 14, following a focal to bilateral tonic–clonic seizure, MRI revealed a left parahippocampal gyrus cavernoma, which was resected. Despite this, seizures persisted under multiple antiseizure medications.

Over time, her seizures evolved to include a rising chest sensation and *déjà‐vu*, followed by impaired awareness, oroalimentary automatisms, and right‐hand dystonia, with postictal headaches and aphasia. Scalp EEG showed right anterior and middle temporal spikes, with rare left anterior temporal spikes. Two habitual seizures were recorded: the first began with a right posterior temporal discharge spreading to the entire right temporal electrodes (Figure 1A), while the second was brief and without significant EEG change. Magnetoencephalography revealed predominantly right temporal spikes with rare independent left temporal discharges. Positron emission tomography (PET) demonstrated bilateral temporo‐parietal hypometabolism and focal hypometabolism at the prior resection site. Neuropsychological testing showed impaired verbal declarative memory with preserved visual memory.

**FIGURE 1:**
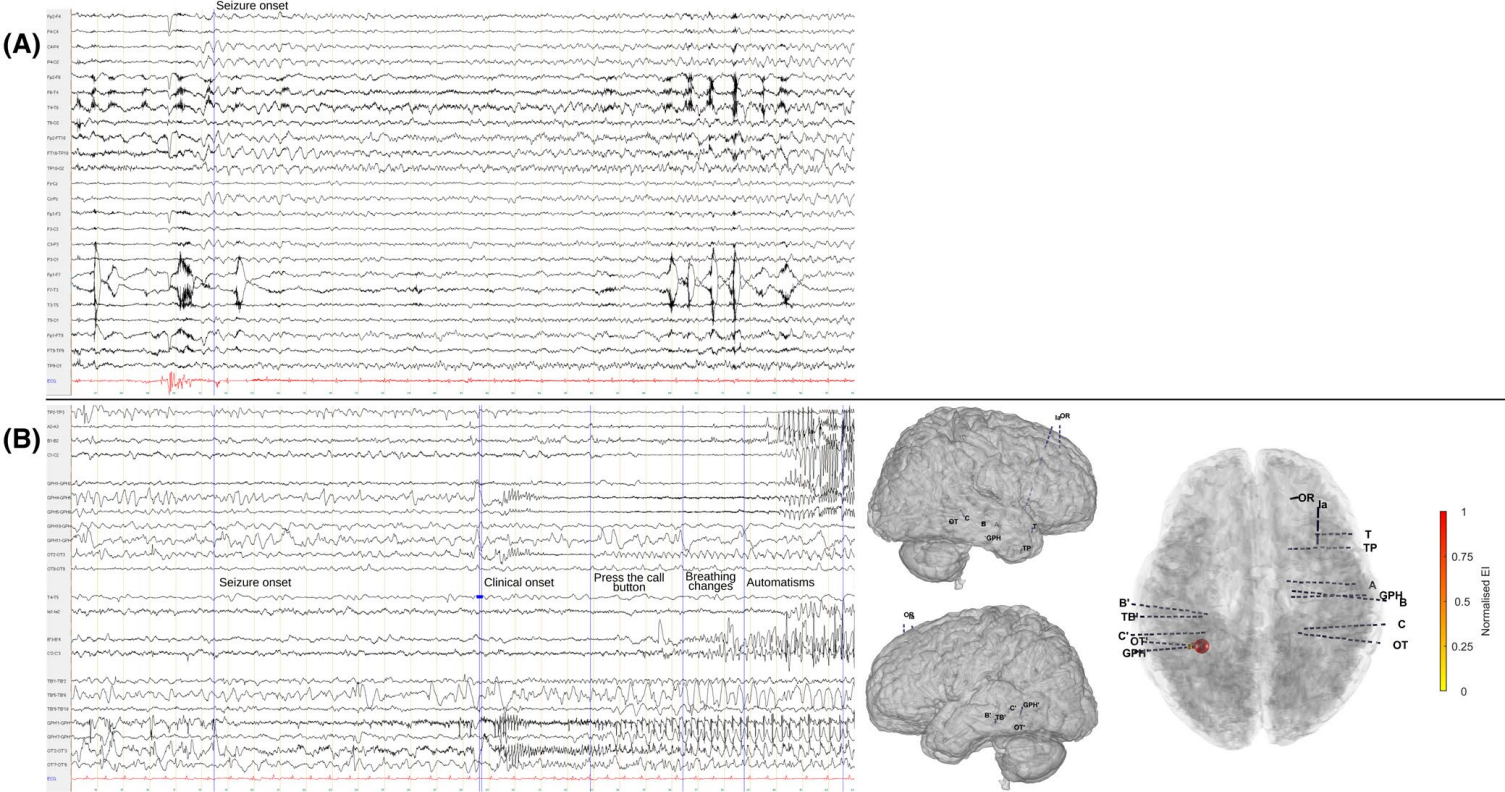
Comparison of scalp EEG (A) and SEEG (B) seizure patterns. Panel A shows a scalp EEG seizure characterized by a right posterior temporal discharge with subsequent propagation to the entire right temporal lobe (high‐pass filter: 0.3 s; low‐pass filter: 70 Hz, epoch length: 30 s, amplitude: 20 μV/mm). Panel B displays SEEG ictal recording marked by initial flattening and disappearance of interictal spikes, followed by a progressive build‐up of low‐voltage fast activity in the left parahippocampal gyrus, and later rhythmic theta activity involving both left and right temporo‐basal regions (high‐pass filter: 0.3 s; epoch length: 30 s, amplitude: 50 μV/mm). A 3D representation of the SEEG electrodes^11^ is provided to illustrate their spatial relationships. SEEG electrode contacts are grouped into five anatomical groups: Right mesial temporal region (TP2–TP3: Temporal pole; A2–A3: Anterior hippocampus; B1–B2: Anterior hippocampus; C1–C2: Posterior hippocampus), right temporo‐basal region (GPH1–GPH2: Parahippocampal cortex; GPH4–GPH5 and GPH5–GPH6: Collateral sulcus; GPH10–GPH11: Anterior inferior temporal gyrus; GPH11–GPH12: Anterior inferior temporal sulcus; OT2–OT3: Lingual sulcus; OT8–OT9: Occipito‐temporal sulcus), right lateral temporal region (T4–T5: Lateral anterior superior temporal gyrus; Ia1–Ia2: Short gyri of the insula), left mesial temporal region (B′3–B′4: Anterior hippocampus; C′2–C′3: Posterior hippocampus), and left temporo‐basal region (TB′1–TB′2: Parahippocampal cortex; TB′5–TB′6: Collateral sulcus; TB′9–TB′10: Anterior inferior temporal gyrus; GPH′1–GPH′2: Collateral sulcus; GPH′7–GPH′8: Posterior inferior temporal gyrus; OT′2–OT′3: Collateral sulcus; OT′7–OT′8: Occipito‐temporal sulcus). The bottom‐right panel displays the quantification of ictal discharges using the epileptogenicity index on 3D representation of the SEEG electrodes. This index evaluates a region's ability to lose background/low‐frequency activity (<12 Hz), to generate high‐frequency ictal discharges (>12 Hz), and the relative earliness of these changes compared with other recorded regions.^12^

Given the discordance between seizure semiology, MRI, and scalp EEG ictal onset, SEEG was performed. It revealed background slowing around the left temporo‐basal resection cavity, as well as in the right temporo‐basal region and temporal pole. Abundant pseudo‐periodic spikes arose from the left temporo‐basal region, sometimes also asymptomatic rhythmic discharges, and fewer spikes were observed in the left entorhinal and parahippocampal gyri. High‐amplitude spikes were also recorded in the right amygdala, hippocampus, and parahippocampal gyrus, activated during sleep and occurring at a lower rate than the left temporo‐basal ones. Habitual seizures were recorded during SEEG, showing a characteristic progression (Figure 1B). They began with flattening and disappearance of interictal spikes, followed by a progressive build‐up of low‐voltage fast activity in the left parahippocampal gyrus, then rhythmic theta activity in both temporo‐basal regions. Two distinct hemispheric patterns were observed: on the left, a sinusoidal alpha rhythm with superimposed fast activity evolved into spike–wave discharges in the temporo‐basal area, propagating to the hippocampus after 15 seconds; on the right, low‐voltage fast activity arose in the temporo‐basal area, spreading to the amygdala and hippocampus after 20 seconds, and to the insula after 25 seconds. Seizure activity terminated earlier on the right than on the left. The patient underwent SEEG‐guided radiofrequency thermocoagulation of the left temporo‐basal contacts surrounding the resection cavity and has remained seizure‐free for 9 months.

This case highlights the key role of anatomo‐electro‐clinical correlations in defining the epileptogenic zone. Any discrepancies should prompt suspicion and warrant further exploration with SEEG.^6^, ^7^ In such cases, bilateral SEEG implantation with extensive sampling of mesial and basal temporal structures is paramount to distinguish delays in regional involvement at seizure onset. In our patient, omission of electrodes around the resection cavity would have led to recording only the bitemporal activity emerging after the true onset, potentially leading to the erroneous diagnosis of bitemporal epilepsy. In our patient, seizure semiology (right‐hand dystonia and postictal aphasia) suggested a left temporal onset, consistent with the left‐sided resection cavity but conflicting with the right‐sided onset on EEG. SEEG exploration demonstrated a left temporo‐basal epileptogenic zone with rapid contralateral propagation. Several factors may explain the false lateralization. The deep location and limited spatial extent of the seizure onset zone, combined with the low amplitude of the initial ictal discharge, likely precluded the detection of early ictal activity on scalp EEG. Consequently, the recorded ictal EEG primarily reflected the propagation of the discharge, which exhibited greater amplitude and spatial spread in the right temporal lobe. Similar mechanisms have been previously proposed in the literature.^1^, ^2^ The resection cavity may also have restricted ipsilateral propagation toward the anterior left temporal lobe, enhancing the visibility of the right‐sided spread.

## CONFLICT OF INTEREST STATEMENT

The authors have no conflict of interest to disclose.

**Test yourself**

1. Which of the following mechanisms can explain false lateralization of ictal EEG onset in temporal lobe epilepsy?
  - Rapid contralateral propagation via commissural pathways
  - High‐amplitude ictal onset in the true seizure zone
  - Low‐amplitude ictal discharges in atrophic or damaged cortex
  - Multiple independent epileptogenic zones
  - Increased scalp EEG sensitivity for mesial temporal structures
2. Which clinical or investigative finding should raise suspicion for false lateralization in a patient with temporal lobe epilepsy?
  - Concordant seizure semiology, imaging, and EEG
  - Ictal EEG onset contralateral to the lesion
  - Bitemporal interictal spikes with consistent unilateral seizure onset on EEG
  - Neuropsychological deficit not concordant with ictal scalp EEG (e.g., verbal memory deficit with right‐sided EEG onset)
  - Discordant semiological lateralizing signs (e.g., dystonic hand)
3. Which of the following strategies helps reduce the risk of misinterpretation due to false lateralization during presurgical evaluation of temporal lobe epilepsy?
  - Use of scalp EEG only
  - Bilateral SEEG implantation with comprehensive sampling of mesial and basal temporal structures
  - Recording of several habitual seizures
  - Relying solely on interictal spike location
  - Avoiding overinterpretation of the frequent increase in mesiotemporal spikes during sleep, which may occur in non‐epileptogenic regions

*Answers may be found in the* *supporting information*

## Supporting information


Data S1:

Data S2:

## Data Availability

The data that supports the findings of this study is available on request from the corresponding author. The data is not publicly available due to privacy or ethical restrictions.
