# Supplementary material for: Pitfall of false localization in basal temporal epilepsy: A clinical vignette
Source: Epileptic Disord. 2025 Oct 21;28(1):191–4. doi: 10.1002/epd2.70121 (PMC12964177; doi:10.1002/epd2.70121)
Supplement: Supplementary file 1 — Data S1: [file EPD2-28-191-s001.pptx]

## Slide 1
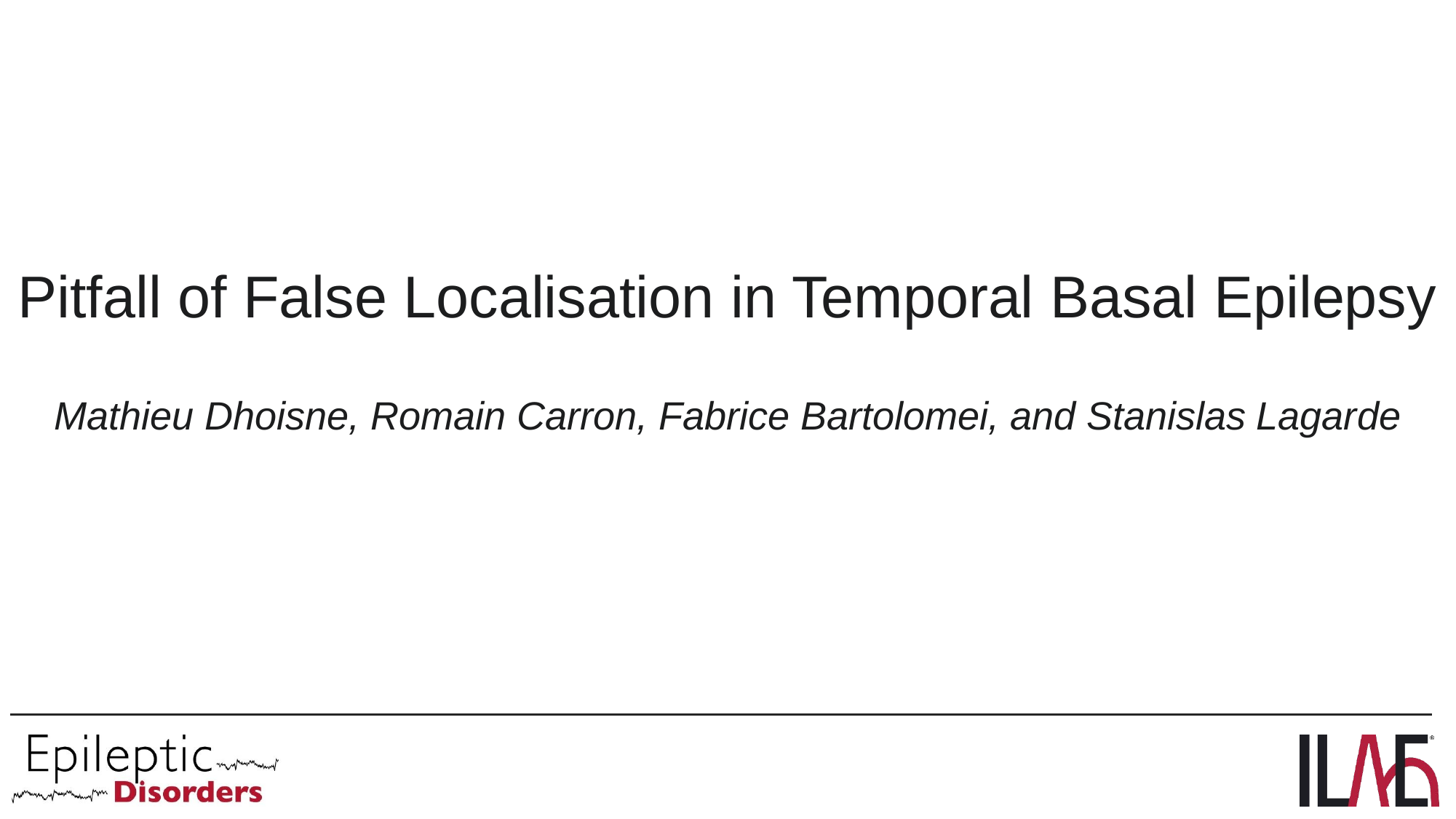

# Pitfall of False Localisation in Temporal Basal Epilepsy
Mathieu Dhoisne, Romain Carron, Fabrice Bartolomei, and Stanislas Lagarde

## Slide 2
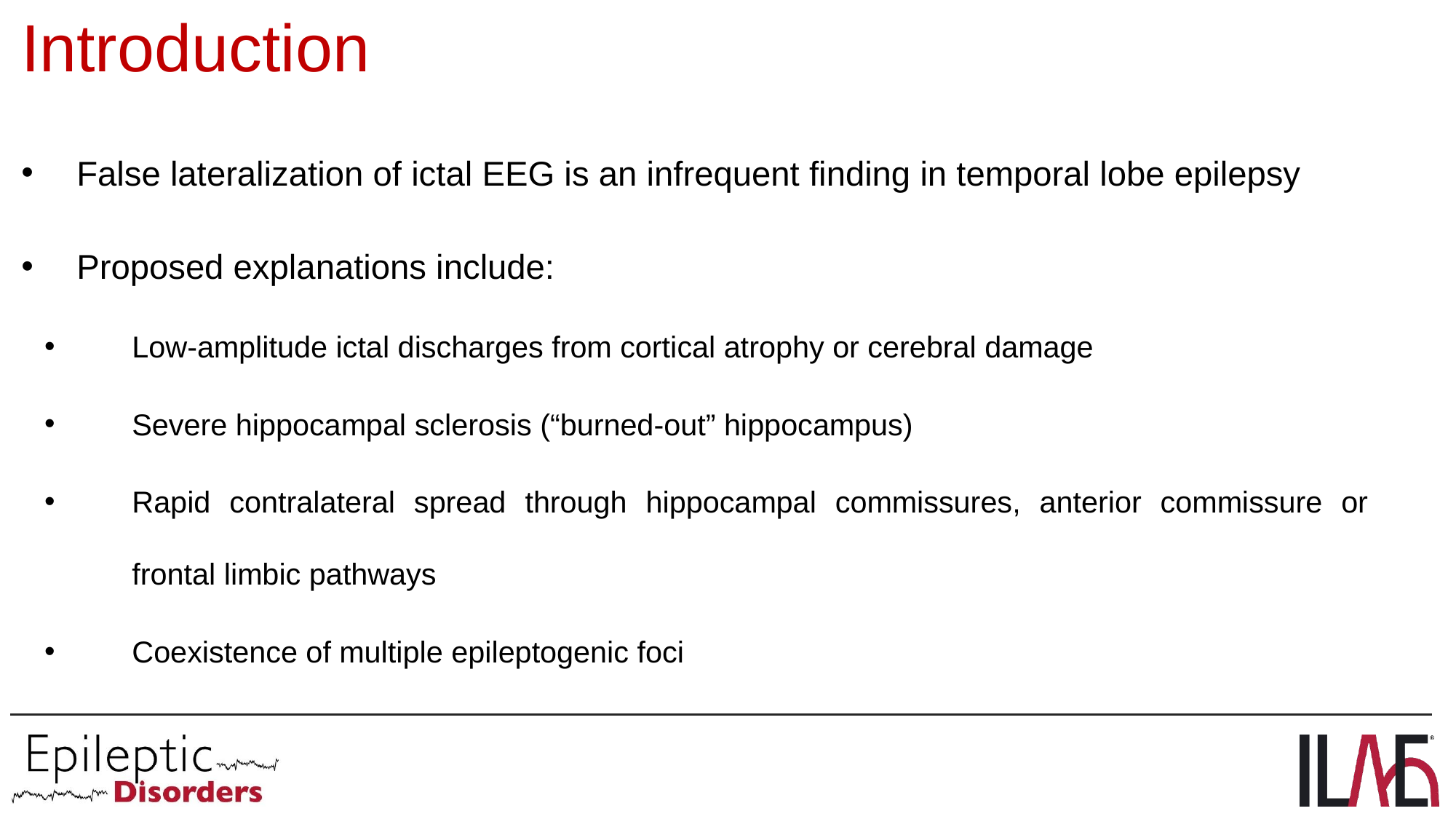

Introduction
False lateralization of ictal EEG is an infrequent finding in temporal lobe epilepsy
Proposed explanations include:
Low-amplitude ictal discharges from cortical atrophy or cerebral damage
Severe hippocampal sclerosis (“burned-out” hippocampus)
Rapid contralateral spread through hippocampal commissures, anterior commissure or frontal limbic pathways
Coexistence of multiple epileptogenic foci

## Slide 3
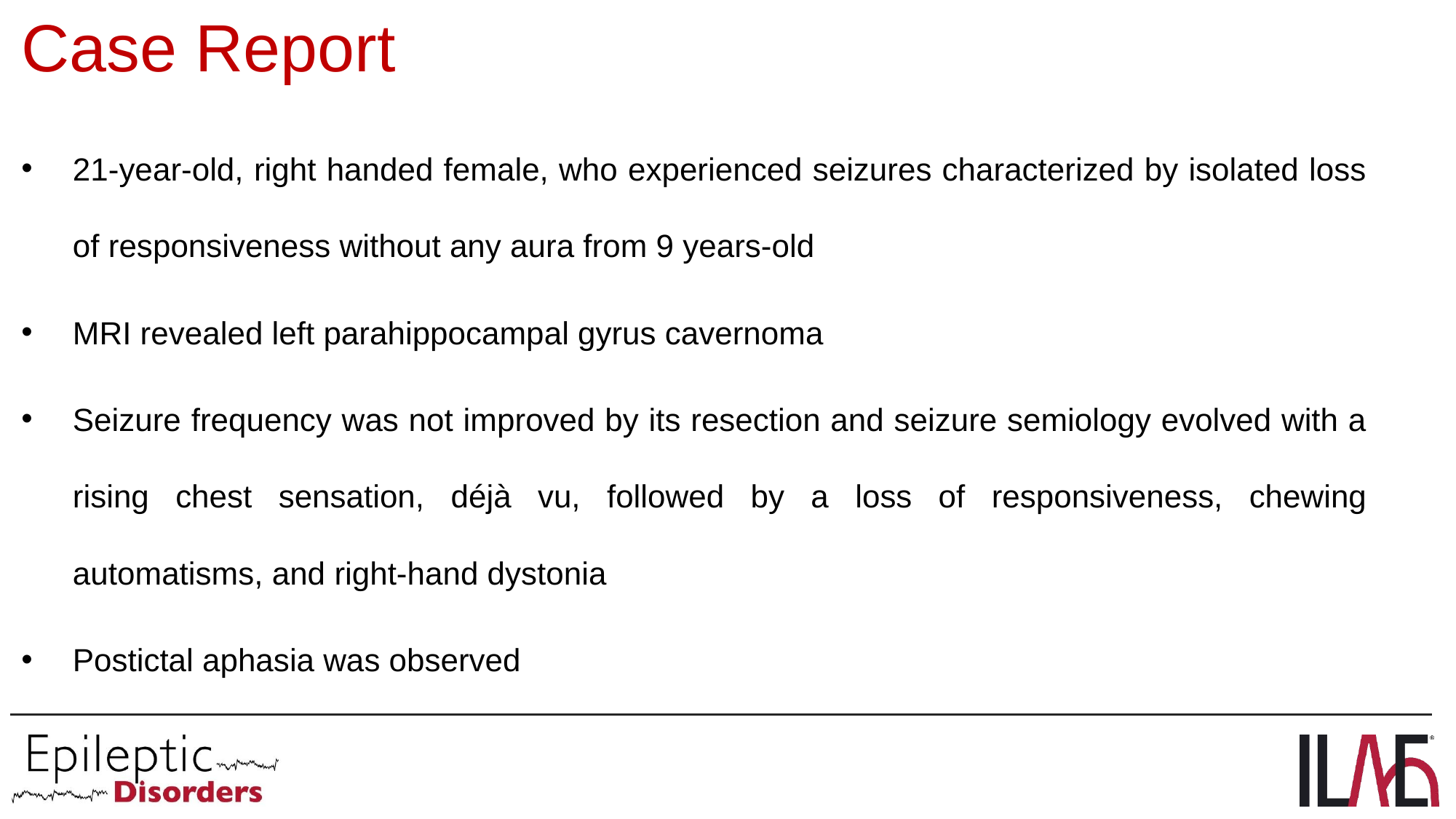

Case Report
21-year-old, right handed female, who experienced seizures characterized by isolated loss of responsiveness without any aura from 9 years-old
MRI revealed left parahippocampal gyrus cavernoma
Seizure frequency was not improved by its resection and seizure semiology evolved with a rising chest sensation, déjà vu, followed by a loss of responsiveness, chewing automatisms, and right-hand dystonia
Postictal aphasia was observed

## Slide 4
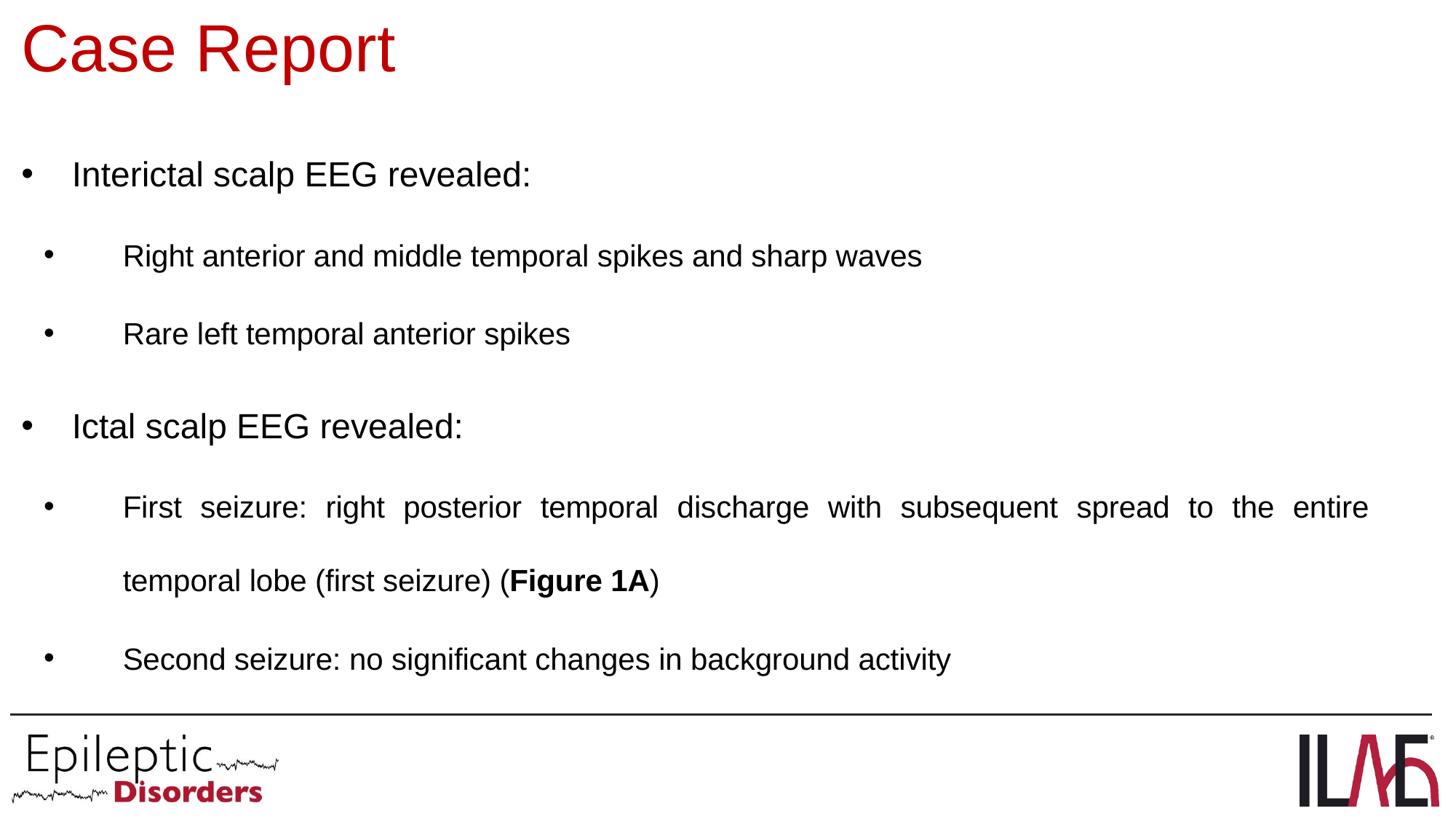

Case Report
Interictal scalp EEG revealed:
Right anterior and middle temporal spikes and sharp waves
Rare left temporal anterior spikes
Ictal scalp EEG revealed:
First seizure: right posterior temporal discharge with subsequent spread to the entire temporal lobe (first seizure) (Figure 1A)
Second seizure: no significant changes in background activity

## Slide 5
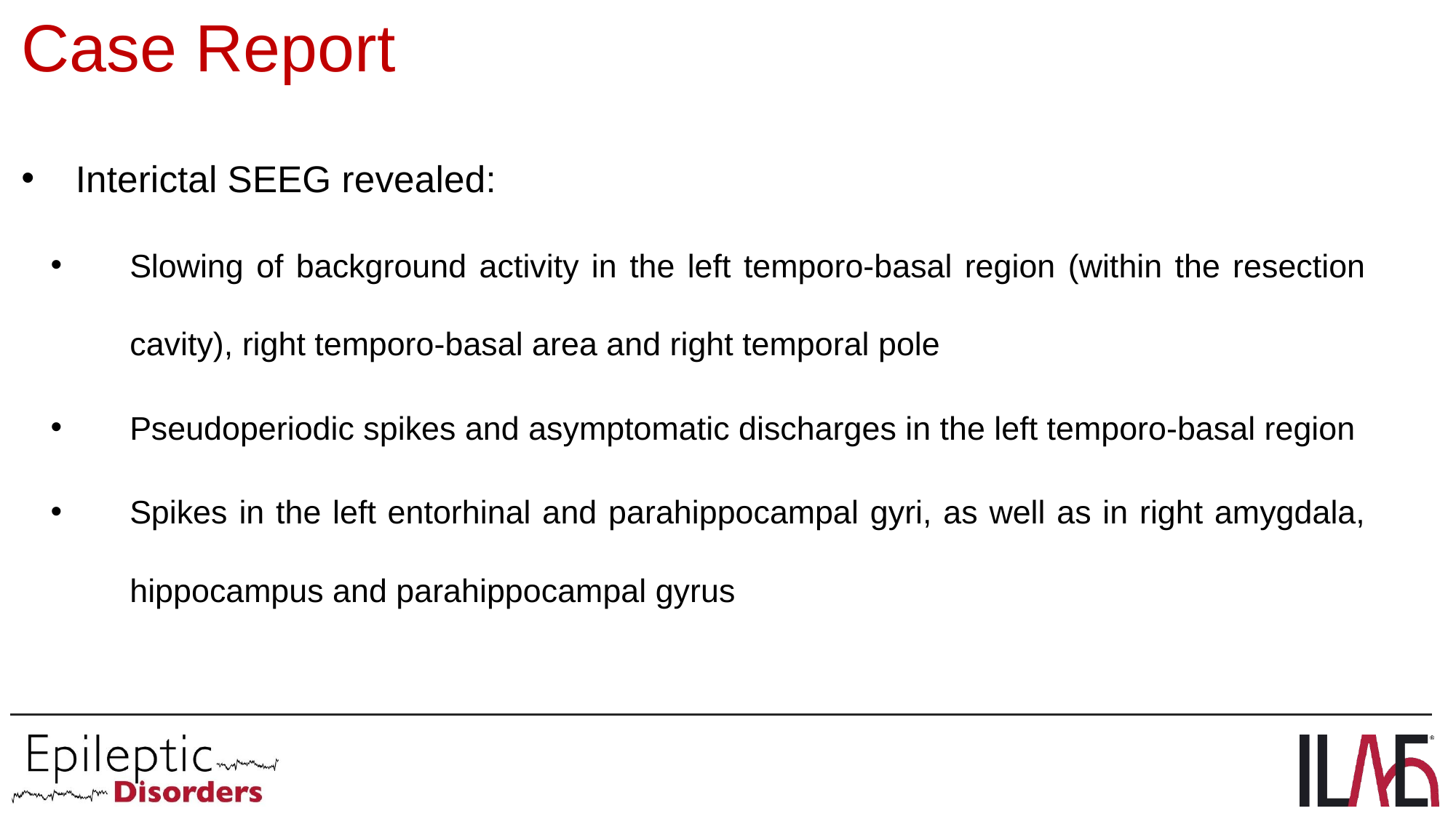

Case Report
Interictal SEEG revealed:
Slowing of background activity in the left temporo-basal region (within the resection cavity), right temporo-basal area and right temporal pole
Pseudoperiodic spikes and asymptomatic discharges in the left temporo-basal region
Spikes in the left entorhinal and parahippocampal gyri, as well as in right amygdala, hippocampus and parahippocampal gyrus

## Slide 6
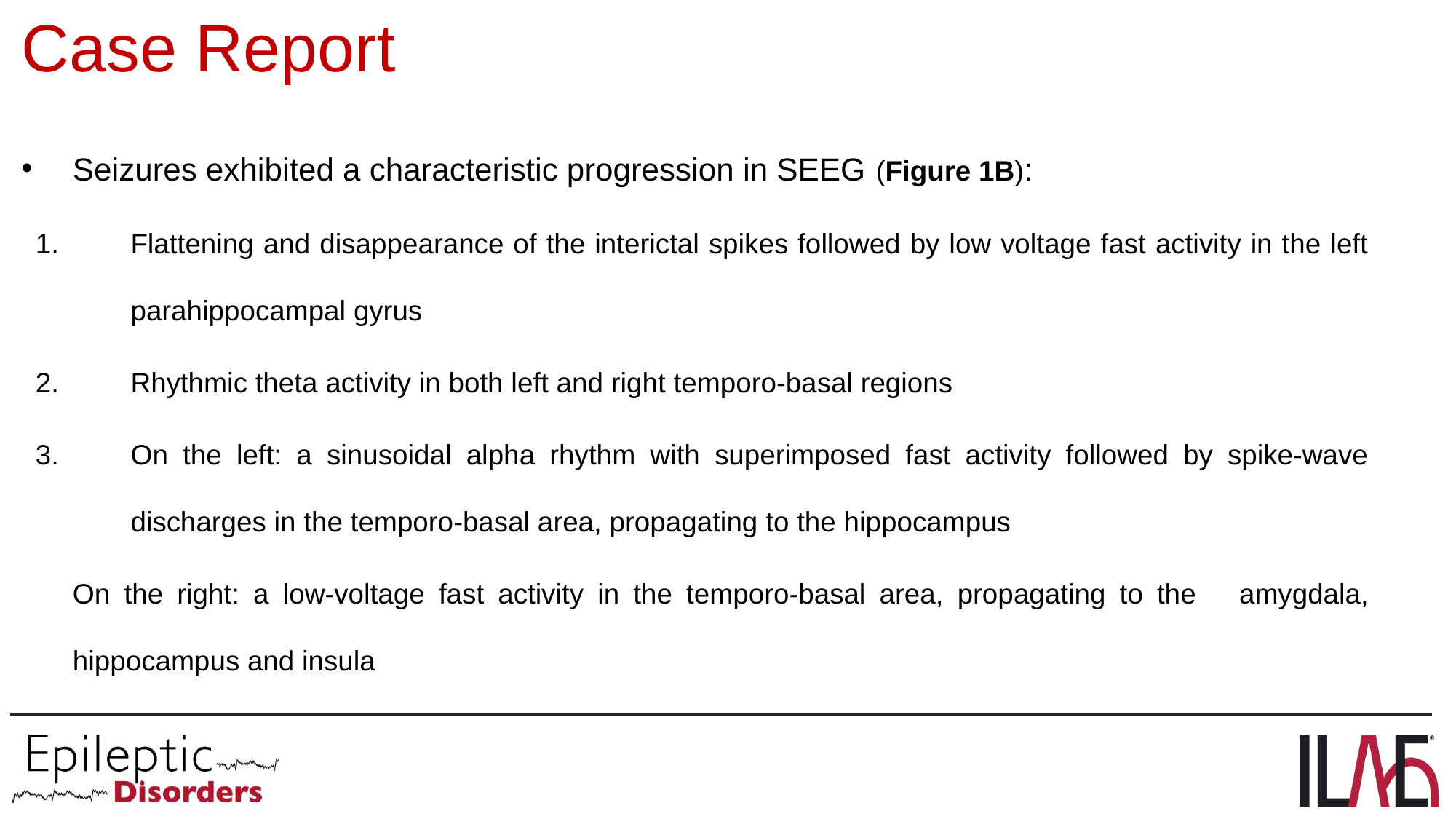

Case Report
Seizures exhibited a characteristic progression in SEEG (Figure 1B):
Flattening and disappearance of the interictal spikes followed by low voltage fast activity in the left parahippocampal gyrus
Rhythmic theta activity in both left and right temporo-basal regions
On the left: a sinusoidal alpha rhythm with superimposed fast activity followed by spike-wave discharges in the temporo-basal area, propagating to the hippocampus
	On the right: a low-voltage fast activity in the temporo-basal area, propagating to the 	amygdala, hippocampus and insula

## Slide 7
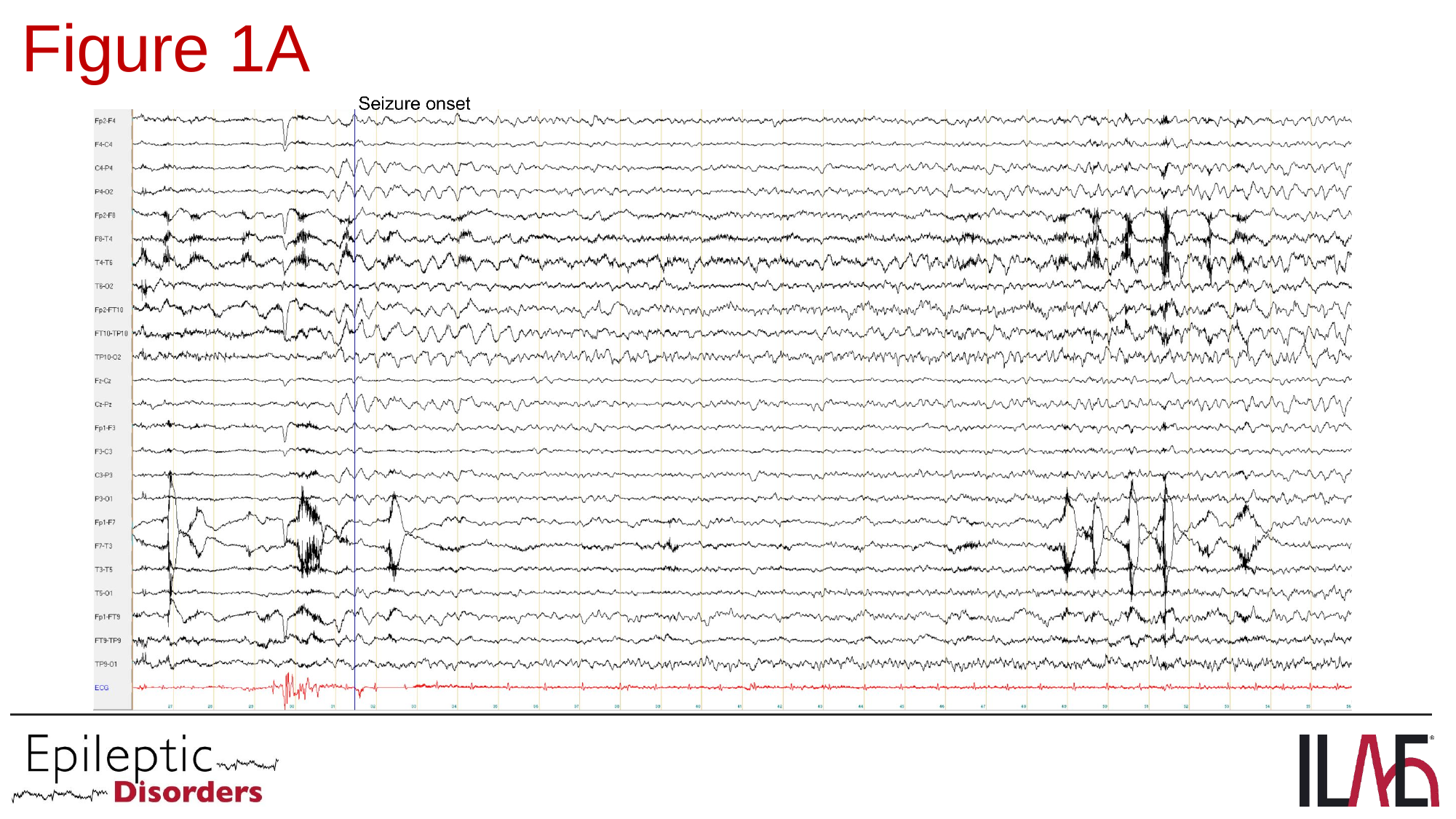

Figure 1A

## Slide 8
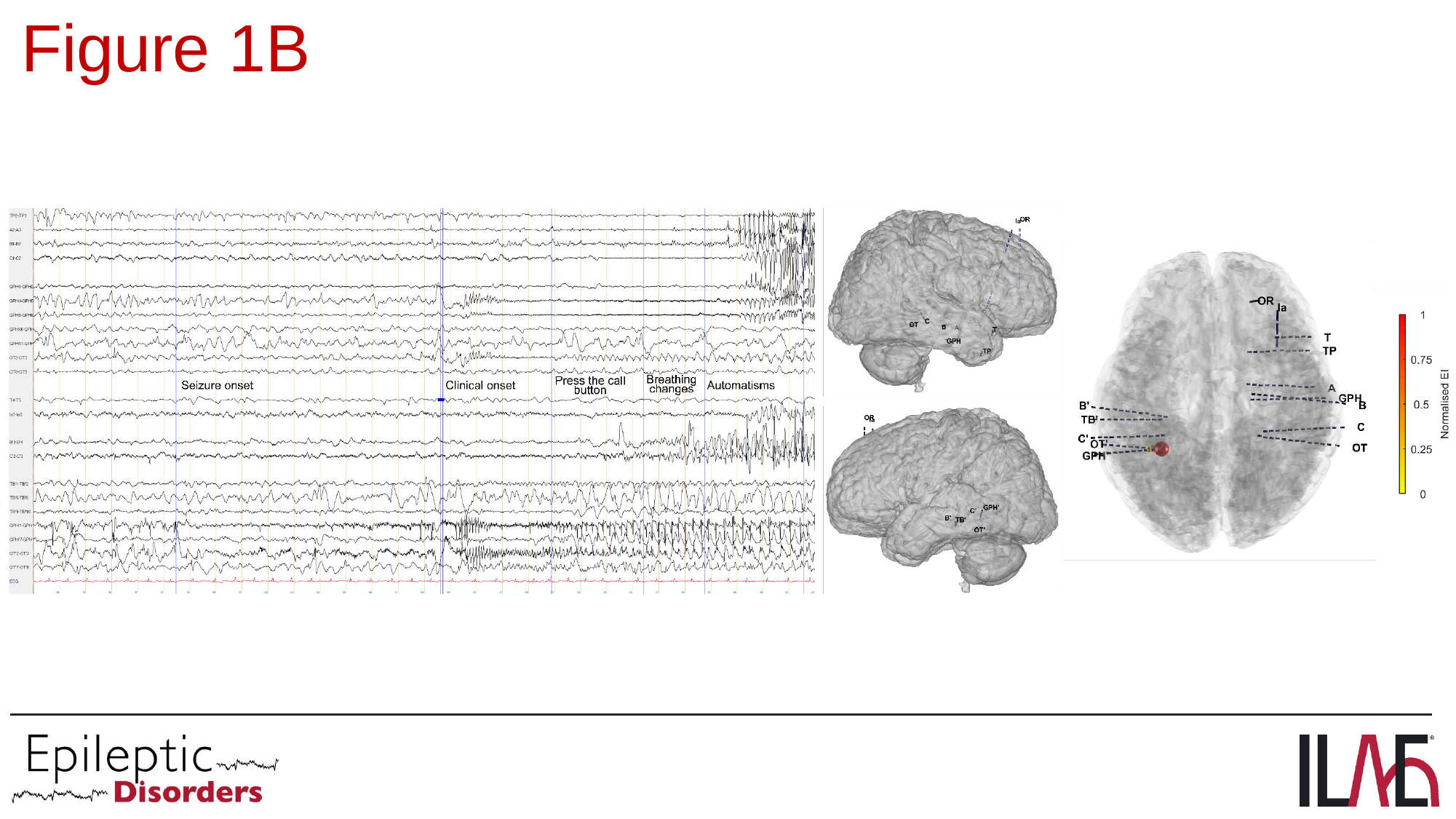

Figure 1B

## Slide 9
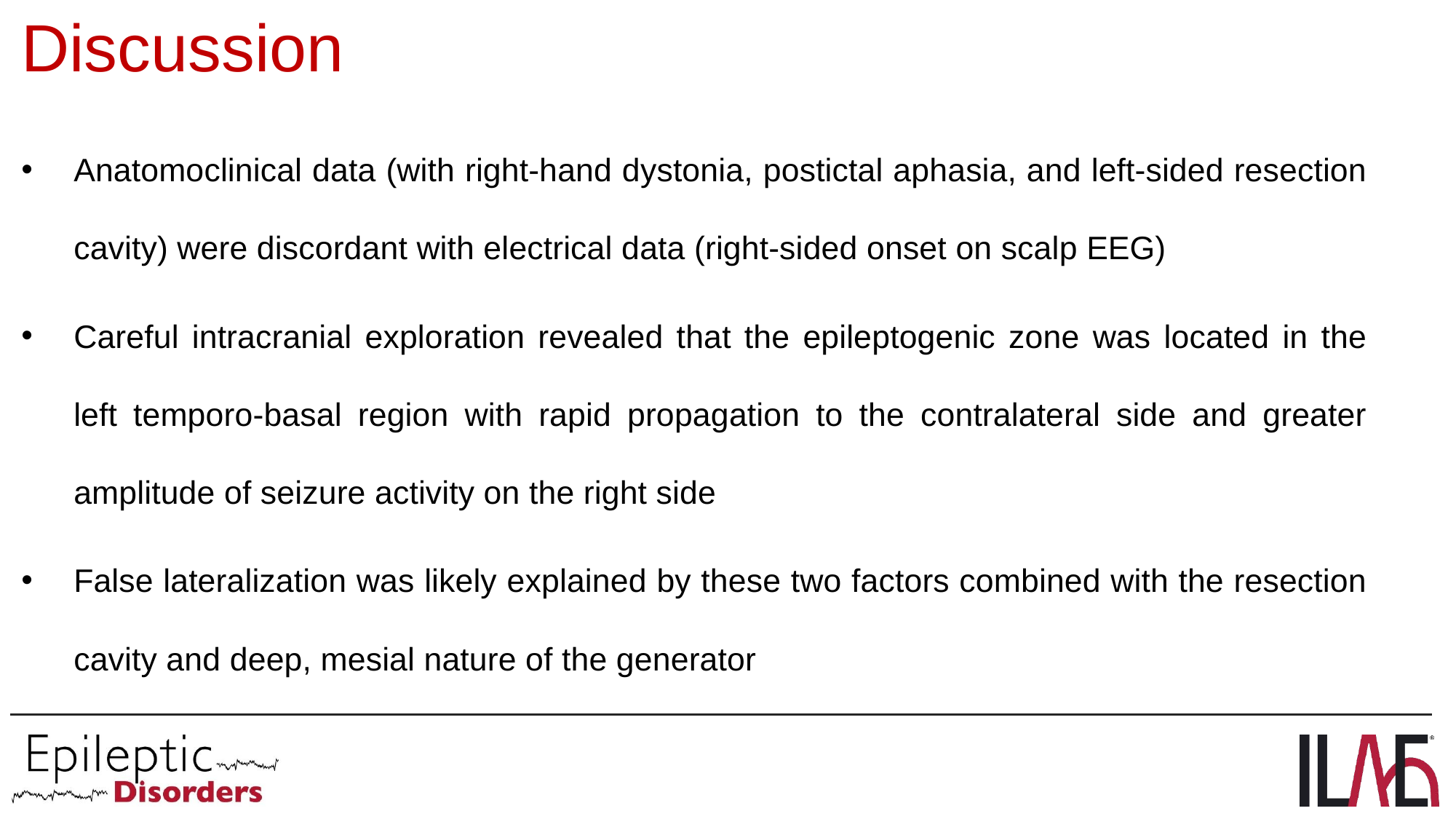

Discussion
Anatomoclinical data (with right-hand dystonia, postictal aphasia, and left-sided resection cavity) were discordant with electrical data (right-sided onset on scalp EEG)
Careful intracranial exploration revealed that the epileptogenic zone was located in the left temporo-basal region with rapid propagation to the contralateral side and greater amplitude of seizure activity on the right side
False lateralization was likely explained by these two factors combined with the resection cavity and deep, mesial nature of the generator

## Slide 10
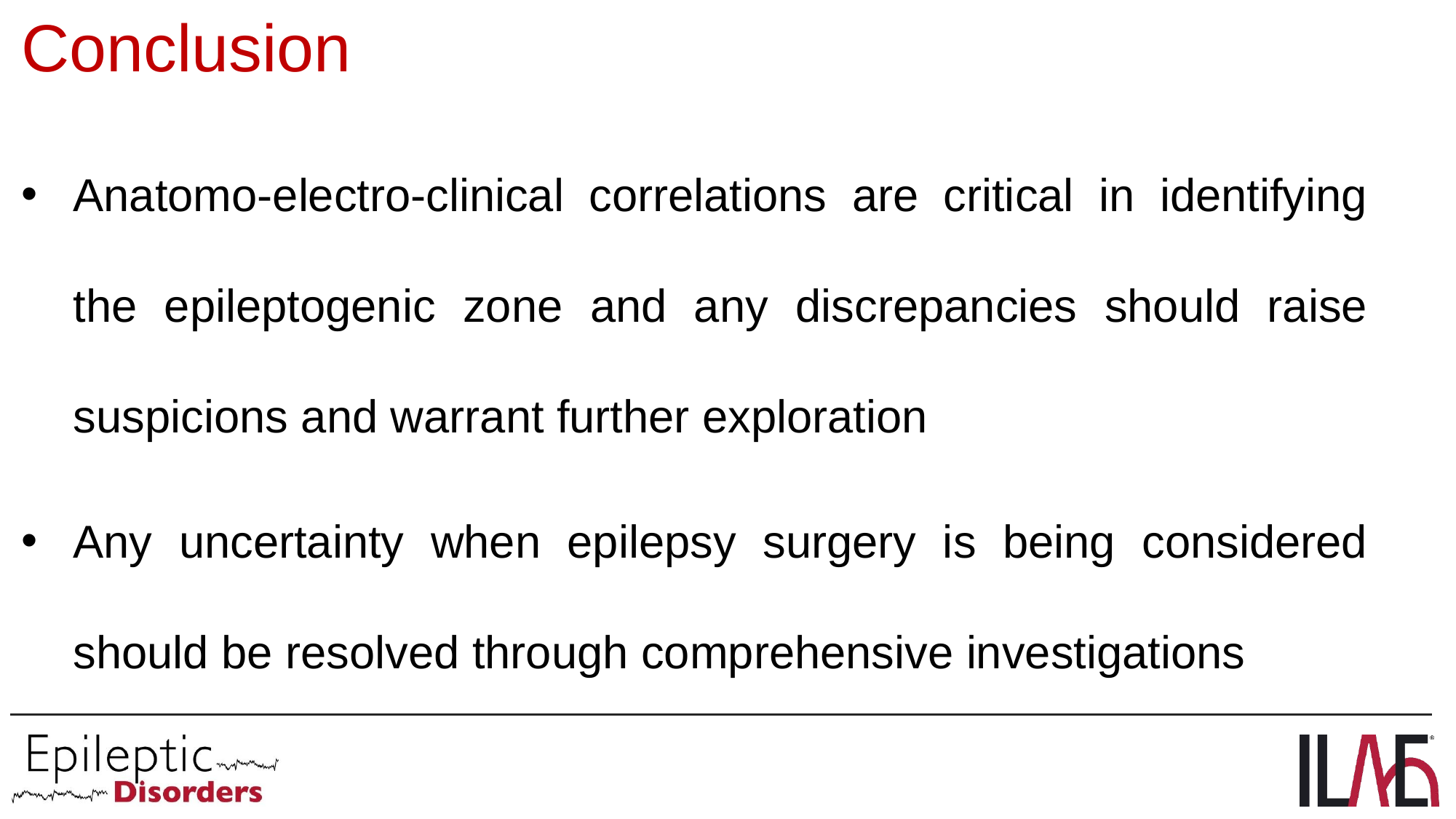

Conclusion
Anatomo-electro-clinical correlations are critical in identifying the epileptogenic zone and any discrepancies should raise suspicions and warrant further exploration
Any uncertainty when epilepsy surgery is being considered should be resolved through comprehensive investigations
