# Supplementary material for: Pitfall of false localization in basal temporal epilepsy: A clinical vignette
Source: Epileptic Disord. 2025 Oct 21;28(1):191–4. doi: 10.1002/epd2.70121 (PMC12964177; doi:10.1002/epd2.70121)
Supplement: Supplementary file 2 — Data S2: [file EPD2-28-191-s002.docx]

**Test yourself Answers:**

1. Correct answers: A, C, D
2. Correct answer: B, D, E
3. Correct answers: B, C, E
